# Allogeneic Transplant for CMML

**DOI:** 10.1007/s11899-025-00754-1

**Published:** 2025-08-11

**Authors:** Nico Gagelmann, Nihar Desai

**Affiliations:** 1 Department of Stem Cell Transplantation, University Medical Center Hamburg-Eppendorf, Hamburg, Germany; 2 Hans Messner Allogeneic Blood and Marrow Transplant Program, Division of Medical Oncology and Hematology, Princess Margaret Cancer Centre, Toronto, Canada; 3 Temerty Faculty of Medicine, University of Toronto, Toronto, Canada

## Abstract

**Purpose of Review:**

Chronic myelomonocytic leukemia (CMML) is a rare hematologic malignancy at the intersection of myelodysplastic (MDS) and myeloproliferative neoplasms, predominantly affecting older adults. Allogeneic hematopoietic cell transplantation (allo-HCT) remains the only curative option, yet its application is limited by the advanced age and comorbidities of most patients. Recent classification updates and refined prognostic tools, particularly molecularly integrated models like CPSS-Mol have enhanced patient stratification and informed transplant timing. The aim of this review is to highlight the evolving landscape of CMML management, with a focus on the role of allo-HCT.

**Recent Findings:**

Novel studies patients demonstrated that individualized transplant timing significantly improved life expectancy. Optimizing transplant outcomes hinges on several factors:managing pretransplant splenomegaly, choosing appropriate debulking strategies, selecting optimal donors, and tailoring conditioning regimens. New data favor treosulfan-based and thiotepa-busulfan regimens for their favorable toxicity and relapse profiles. Post-transplant, strategies like post-transplant cyclophosphamide (PTCy) for GVHD prophylaxis and emerging approaches to minimal residual disease (MRD) monitoring offer additional refinements in patient management. While no MRD studies are CMML-specific, extrapolation from MDS supports its role in relapse prediction. Innovative therapies, including hypomethylating agent combinations, venetoclax, targeted inhibitors, and immunotherapies are under active investigation, with potential to improve pre- and post-transplant outcomes.

**Summary:**

Advancements in molecular classification, dynamic prognostic tools, and therapeutic strategies are reshaping the CMML treatment paradigm. Personalized approaches that integrate genetic risk, patient fitness, and disease characteristics are enabling more effective transplant strategies, with the ultimate goal of extending survival and improving quality of life in this complex and historically difficult-to-treat malignancy.

## Introduction

Chronic myelomonocytic leukemia (CMML) is a rare and biologically complex hematologic malignancy that shares features of both myelodysplastic syndromes (MDS) and myeloproliferative neoplasms (MPNs) [1]. It primarily affects older adults, with a median age at diagnosis ranging from 70 to 75 years, and accounts for approximately 15–20%of all MDS/MPN overlap syndromes. CMML is characterized by a clonal proliferation of hematopoietic stem and progenitor cells, leading to sustained peripheral blood monocytosis (≥ 0.5 × 10/L) and bone marrow dysplasia [2, 3]. The clinical presentation is highly variable, reflecting the diverse genetic and molecular landscapes underlying the disease [4]. Patients can present with either a myelodysplastic phenotype, characterized by cytopenias and ineffective hematopoiesis, or a myeloproliferative phenotype, marked by leukocytosis, splenomegaly, and constitutional symptoms such as fever, weight loss, and night sweats [5].

The pathogenesis of CMML is driven by a complex interplay of genetic, epigenetic, and environmental factors. Key mutations frequently implicated in CMML include *ASXL1*,* TET2*,* SRSF2*,* RUNX1*, and *NRAS*, which contribute to clonal hematopoiesis and disease progression [6]. Notably, *ASXL1* mutations are associated with a particularly poor prognosis, while *TET2* mutations, when present without *ASXL1* co-mutation, are linked to a more indolent disease course [7]. The combination of these mutations and additional genetic lesions can lead to progressive bone marrow failure and transformation to acute myeloid leukemia (AML), a common cause of death in patients with CMML [8, 9]. Recent insights into the role of clonal hematopoiesis of indeterminate potential (CHIP) and age-related clonal hematopoiesis (ARCH) have further highlighted the importance of early mutational events in CMML pathogenesis, highlighting the need for early detection and intervention [10].

Despite advances in supportive care and targeted therapies, allogeneic stem cell transplantation (allo-HCT) remains the only potentially curative option, offering the possibility of long-term disease-free survival through the eradication of the malignant clone and restoration of normal hematopoiesis [11, 12]. However, the decision to proceed to allo-HCT is complex, given the advanced age, frequent comorbidities, and the substantial risks associated with the procedure, including graft-versus-host disease (GVHD) and non-relapse mortality (NRM). For carefully selected patients, particularly those with high-risk disease and adverse molecular profiles, allo-HCT represents the most effective approach to achieving durable remission and potential cure.

### Epidemiology

CMML is relatively rare, with an estimated annual incidence of 0.3 to 0.4 cases per 100,000 individuals, accounting for 10–20%of MDS/MPN overlap syndromes [13]. Its rarity has historically limited robust clinical trial data, necessitating careful extrapolation from related diseases.

### Classification of CMML

The recently updated International Consensus Classification (ICC, 2022) and the 2022 World Health Organization (WHO) classification have introduced similar modifications to the diagnostic criteria for CMML [14, 15]. These updates include the elimination of the CMML-0 subtype, a reduction in the monocyte threshold to ≥ 0.5 × 10^9^/L, and a continued emphasis on distinguishing between myelodysplastic and myeloproliferative subtypes. Despite these changes, both systems have maintained the same blast count thresholds and, as a result, can be used interchangeably for CMML diagnosis. However, neither classification currently incorporates subtyping based on specific mutational signatures [16].

Notably, both the ICC and WHO classifications have revised the criteria for AML to account for specific genetic mutations. For example, AML with *NPM1* mutations is now defined without a minimum blast count threshold (WHO 2022), while AML with *CEBPA* mutations requires at least 10%blasts (ICC 2022). Given this, patients with CMML harboring these specific mutations should be considered and managed as AML, regardless of blast percentage.

Additionally, the ICC 2022 has introduced a new disease category, MDS/AML, characterized by 10–19%blasts and the presence of high-risk mutations such as *TP53*,* ASXL1*,* BCOR*,* EZH2*,* RUNX1*,* SF3B1*,* STAG2*,* U2AF1*, or *ZRSR2*. This category acknowledges the overlapping features of MDS and AML in certain patients but does not currently extend to CMML, which remains classified separately despite potential molecular overlaps.

### Risk Stratification for Identifying Transplant Candidates

**Fig. 1:**
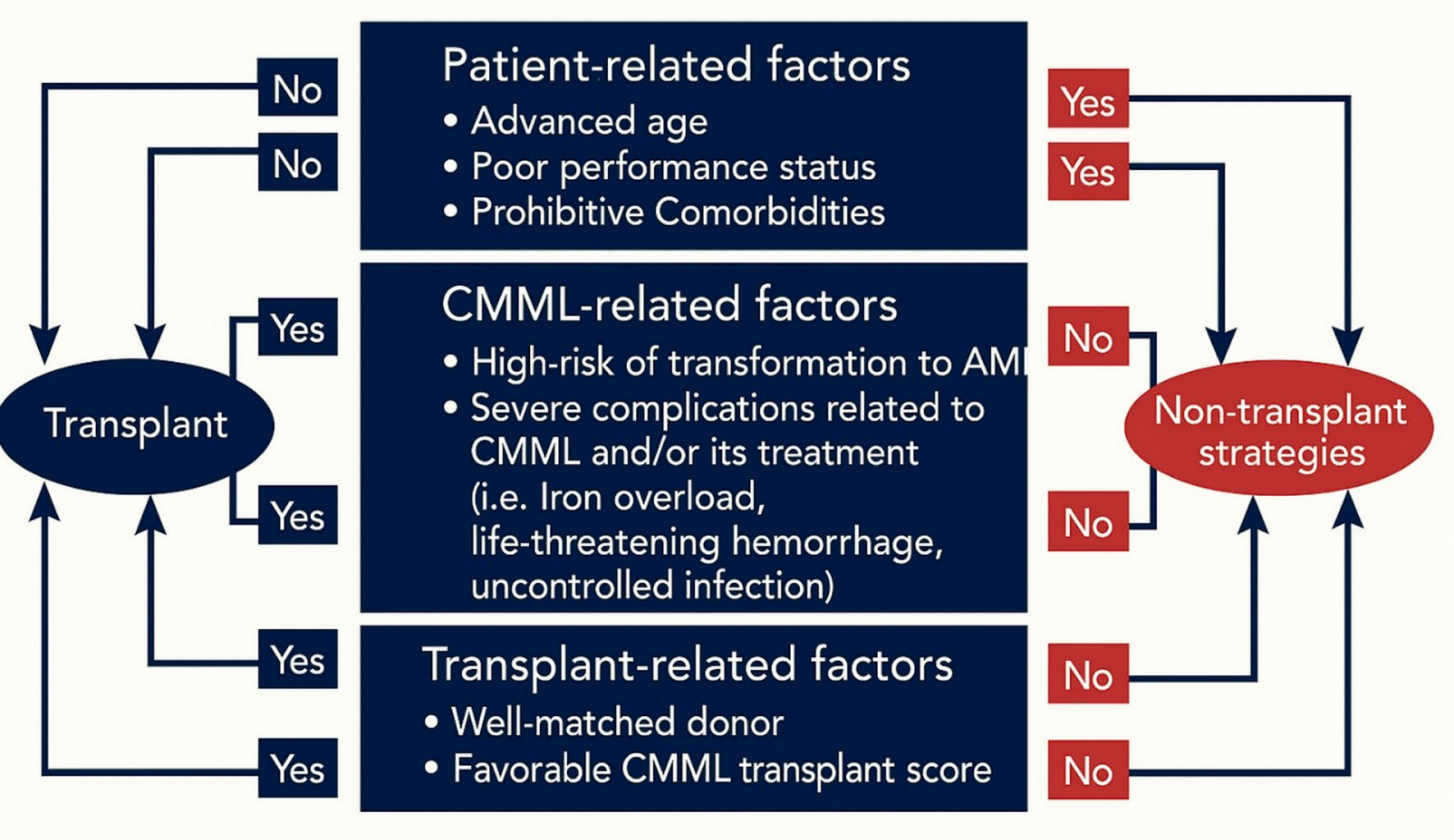
Factors influencing decision for transplant or no transplant

Effective risk stratification is critical for determining which patients with CMML are most likely to benefit from allo-HCT [7, 17–20]. Selecting appropriate transplant candidates involves balancing the potential for long-term remission against the significant risks of transplantation, including GVHD and NRM (Fig. 1) [21].

Historically, the prognostic models used to stratify CMML patients were adapted from MDS scoring systems, including the International Prognostic Scoring System (IPSS) and its revised version (IPSS-R). Over time, more CMML-specific tools have been developed, such as the Düsseldorf score, MD-Anderson Prognostic Score, modified Bournemouth score, CMML-specific Prognostic Scoring System (CPSS), and the Mayo model. These models, while broadly effective, often lacked incorporation of molecular data, which has become increasingly relevant for predicting disease progression and overall survival [18, 22].

In recent years, molecularly integrated scoring systems have been introduced to improve prognostic accuracy. These include the Groupe Francophone des Myelodysplasies score (GFM, focused on *ASXL1* mutations), Mayo Molecular Model (MMM), and the CPSS-Molecular (CPSS-Mol), which considers mutations in *ASXL1*,* RUNX1*,* NRAS*, and *SETBP1*. The European LeukemiaNet (ELN) and 2018 European Hematology Association (EHA) guidelines currently recommend five key scoring systems for CMML, including the MD-Anderson Prognostic Score, CPSS (in the absence of molecular data), GFM, MMM, and CPSS-Mol when molecular data is available.

While these newer molecular models offer significant improvements, it is important to recognize that many of them were developed based on untreated historical cohorts and have not been extensively validated for predicting transplant-specific outcomes. Recent retrospective analyses have suggested that IPSS-M, for instance, correlates well with post-transplant outcomes in MDS [23, 24], indicating its potential applicability to CMML. Similarly, the CPSS-Mol has demonstrated superior predictive power over traditional scoring systems in identifying patients likely to benefit from early transplantation, as shown in studies involving large CMML transplant cohorts [25].

Most recently, a study developed a simple, clinically based survival model for CMML, called BLAST, using 457 molecularly annotated patients [18]. The model incorporates circulating blasts ≥ 2%, leukocytes ≥ 13 × 10⁹/L, and anemia severity, stratifying patients into low (0 points), intermediate (1 point), and high-risk (2–4 points) groups with median overall survival of 63, 28, and 13 months, respectively. BLAST showed strong predictive accuracy (AUC 0.77/0.85 at 3/5 years), comparable to existing molecular models. Unfavorable mutations (e.g., *DNMT3A*,* ASXL1*,* TP53*) [26] and favorable ones (e.g., *TET2*,* PHF6*) were identified and incorporated into a combined clinical-molecular model, BLAST-mol, improving performance (AUC 0.80/0.86). Both models were validated in external cohorts. Risk factors for leukemic transformation included adverse mutations, leukocytosis, and elevated blast counts. BLAST and BLAST-mol offer accessible, effective tools for global CMML risk stratification.

However, applying these risk models to real-world clinical decision-making remains complex. For patients with lower-risk CMML, the immediate risks associated with allo-HCT may outweigh potential long-term benefits, as these patients can often have prolonged survival without early intervention. In contrast, patients with high-risk features, including adverse genetic mutations or elevated blast percentages, are more likely to benefit from early transplantation, as delaying allo-HCT in this group can lead to disease progression, loss of transplant eligibility, and worse overall outcomes.

**Fig. 2:**
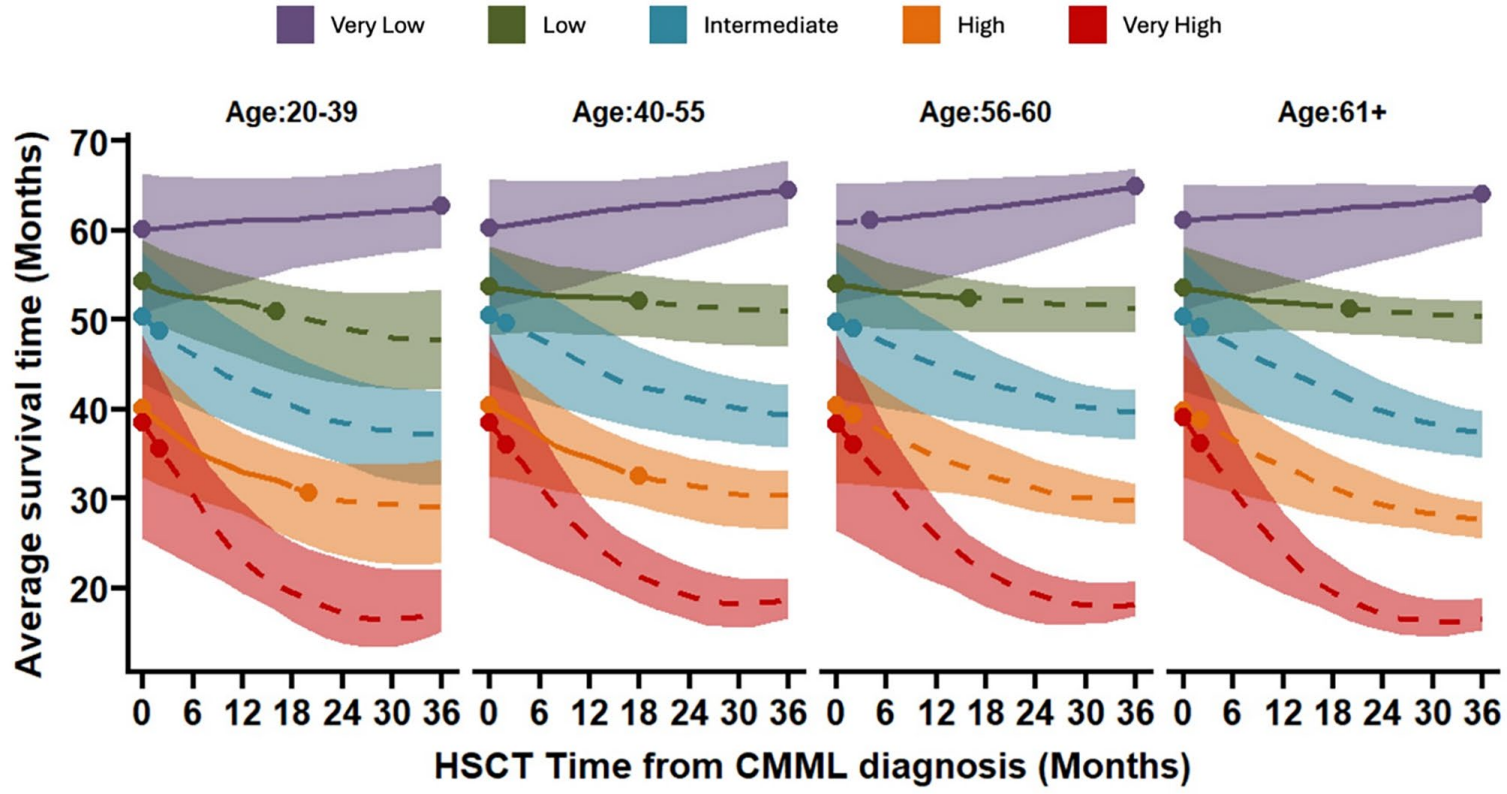
Benefit of timing of HCT according to age

A most recent retrospective study analyzed a large international cohort of 3,182 CMML patients, including 769 (24%) who underwent allo-HCT [27]. To evaluate the impact of different transplant timing strategies, the researchers constructed flexible parametric survival models to assess key transition hazards, including the risk of AML transformation, non-transplant mortality, post-transplant relapse, and death without disease recurrence. These models incorporated patient age and various prognostic scoring systems (CPSS, CPSS-Mol, and iCPSS) as explanatory variables. A semi-Markov multi-state model based on microsimulation was then developed to estimate optimal transplant timing, using Restricted Mean Survival Time (RMST) over an 8-year period to compare different stratification strategies. The analysis revealed that patients classified as very low or low risk by the iCPSS (*n* = 1998, 62%) had better outcomes with delayed transplantation (24–36 months after diagnosis). In contrast, those in the intermediate, high, and very high-risk groups (*n* = 1184, 38%) benefited from early transplantation (3–6 months after diagnosis), resulting in longer RMST. Notably, the iCPSS approach led to a change in recommended transplant timing for 31%and 35%of patients compared to CPSS and CPSS-Mol, respectively. Specifically, 726 patients (22%) who would have been advised to delay allo-HCT under the CPSS strategy were found to benefit from immediate transplantation under the iCPSS model, while 855 patients (26%) identified as immediate HSCT candidates by CPSS-Mol actually had better outcomes with delayed transplantation. Overall, these shifts in transplant timing strategies resulted in significant gains in life expectancy, averaging 1.2 to 1.4 years, depending on the risk model used (*p* < 0.01). The findings highlight the value of integrating clinical and molecular data into transplant decision-making for CMML. The iCPSS-based Decision Support System effectively refines risk stratification and individualizes transplant timing, offering a data-driven alternative to traditional clinical judgment. This personalized approach significantly improves patient outcomes, demonstrating the potential of iCPSS to guide more precise, life-extending transplantation strategies (Fig. 2).

### Risk Stratification for Predicting Post-Transplant Outcomes

While the CPSS and CPSS-Mol scoring systems are commonly used to assess relapse risk and overall survival in CMML patients undergoing allo-HCT, they have notable limitations when it comes to predicting post-transplant outcomes [19, 20]. These models, primarily designed to guide initial disease prognosis, may not fully capture the complex factors influencing post-HCT survival, as they were not specifically developed for the transplant setting. Importantly, using these scores dynamically at the time of transplant, rather than relying solely on initial diagnosis assessments, may offer more accurate predictions of post-transplant outcomes. However, this approach remains largely unvalidated and requires further clinical research.

Additionally, certain high-risk genetic mutations, such as *TP53*, are known to significantly impact post-transplant survival but are not comprehensively included in current molecularly integrated scores like the CPSS-Mol [28–31]. This highlights a critical gap in existing models, emphasizing the need for more sophisticated, transplant-specific risk stratification tools.

To address these limitations, several dedicated transplant-specific prognostic models have been developed. One example is the CMML-specific transplant score, which was designed and validated in a cohort of 240 CMML patients undergoing allo-HCT [25]. This model integrates both molecular data (e.g., *ASXL1* and *NRAS* mutations) and clinical parameters (such as bone marrow blast percentage and comorbidity index) to provide a more precise post-transplant risk assessment. It stratifies patients into five distinct risk groups, with 5-year survival rates ranging from 81%for the lowest-risk group to 19%for the highest-risk group, and non-relapse mortality rates spanning from 5 to 51%. This model has demonstrated superior predictive performance compared to more generalized tools like the CPSS-Mol, which were not specifically designed for transplant patients.

Other transplant-focused tools have also been proposed, including the endothelial activation and stress index (EASIX), which aims to predict NRM by assessing markers of endothelial damage. However, some of these newer models have faced criticism for not being realizable in every day clinical practice by being too complex to calculate and including factors like GVHD without appropriately accounting for inherent statistical biases, underscoring the need for careful model refinement [32, 33].

To further improve predictive accuracy, future transplant-specific scoring systems may need to incorporate additional variables such as donor type, stem cell source, and conditioning regimen intensity. These factors, which significantly influence transplant outcomes, could enhance the precision of risk assessment and better guide personalized post-transplant care.

### Pretransplant Management of Splenomegaly

Splenomegaly is a common finding in patients with CMML, often reflecting the underlying myeloproliferative component of the disease. In many cases, splenomegaly is mild and can be managed conservatively without specific intervention. However, a subset of patients presents with massive splenomegaly, which can significantly complicate the transplant process. Large spleens are associated with delayed neutrophil and platelet engraftment, increased transfusion requirements, and higher NRM.

Splenectomy is one approach for managing severe splenomegaly before allo-HCT, as it can improve hematopoietic recovery by reducing splenic sequestration of blood cells and enhancing donor cell engraftment [34]. Studies in myelofibrosis have shown that splenectomy can significantly accelerate neutrophil and platelet recovery. However, splenectomy is a high-risk procedure in the context of CMML, with reported perioperative morbidity rates of approximately 43%and mortality rates around 13%, making this option suitable only for carefully selected patients. For those who avoid splenectomy, it is important to note that spleen size often gradually decreases after successful engraftment, reflecting disease control.

Splenic irradiation presents a less invasive alternative to splenectomy, offering the potential to reduce spleen volume and control disease symptoms prior to transplantation [35]. However, this approach carries its own set of challenges. Splenic irradiation can induce severe, prolonged pancytopenia, which may complicate the transplant process if not carefully managed. Given this risk, it is generally recommended that splenic irradiation be used as an adjunct to conditioning regimens, where the pancytopenia it induces can be mitigated by rapid donor engraftment. Splenic irradiation is associated with reduced relapse risk and similar NRM compared to splenectomy, and appears as much more attractive approach. However, more studies particularly in CMML are needed.

For patients in whom splenectomy or splenic irradiation are deemed too risky, medical therapies such as JAK2 inhibitors may offer an alternative means of controlling splenomegaly. These agents, including ruxolitinib, have been shown to reduce spleen size and improve disease-related symptoms in myelofibrosis and may have a role in selected CMML patients, particularly those with JAK2 mutations or proliferative disease features [36]. However, their use in the pretransplant setting remains investigational, and further studies are needed to define their optimal role in this context.

### Pretreatment

The role of pretransplant treatment, often referred to as debulking, remains a topic of debate in CMML. The primary goal is to reduce the disease burden before allo-HCT, typically by lowering bone marrow blast percentages or achieving complete remission (CR). However, it is still unclear whether this approach significantly improves transplant outcomes in patients with CMML. While some studies suggest that reducing bone marrow blasts to less than 2–10%before transplantation might improve outcomes, this has not been consistently demonstrated, and the impact of achieving CR without minimal residual disease (MRD) negativity remains uncertain [12, 37–39].

The use of hypomethylating agents and venetoclax may be good for debulking but in limited retrospective series has shown no OS benefit. Retrospective studies have provided mixed results regarding the effectiveness of different pretransplant strategies. Some analyses indicate that HMA, such as azacitidine and decitabine, may offer better outcomes than intensive chemotherapy (IC) in certain subgroups [40], including patients with higher-risk disease or those with elevated bone marrow blast counts. However, other studies have found no significant difference in survival or relapse rates between HMA-based and IC-based pretransplant approaches. Importantly, several of these studies included patients who ultimately did not proceed to transplant due to disease progression or treatment-related toxicity, potentially skewing the results [41, 42].

Randomized prospective trials comparing pretransplant strategies in CMML are lacking, making it challenging to establish definitive guidelines. Nevertheless, HMAs remain a commonly used bridging therapy due to their relatively favorable toxicity profile and potential to stabilize disease until a transplant is feasible. However, it should be noted that up to 13–36%of patients who initiate HMA therapy with the intention of proceeding to transplant may never reach transplant due to disease progression, adverse events, or the development of new comorbidities.

A randomized phase III trial compared decitabine and hydroxyurea in 170 patients with newly diagnosed, advanced CMML (MP-CMML). While decitabine led to a significantly higher response rate (63%vs. 35%, *P* = 0.0004), it did not translate into improved event-free survival, which remained similar between the two arms (12 months for decitabine vs. 10 months for hydroxurea;*P* = 0.27). Median OS was also not significantly different (18 vs. 22 months;*P* = 0.67), and the duration of response was comparable. Notably, decitabine reduced the risk of disease progression or transformation to AML (HR 0.62;*P* = 0.005), suggesting some disease-modifying activity. However, this came with a trade-off:a non-significant trend toward increased death without progression (HR 1.55;*P* = 0.04), possibly reflecting treatment-related toxicity or frailty in this older population (median age 72–74). This study underscores a central challenge in MP-CMML:while decitabine may delay transformation, it does not improve long-term survival or event-free outcomes compared to hydroxyurea. Hydroxyurea remains a standard cytoreductive therapy but lacks disease-modifying impact. Thus, beyond cytoreduction, we currently lack effective disease-modifying therapies for MP-CMML. These findings highlight an urgent need for novel approaches that meaningfully alter disease trajectory without adding toxicity, especially for older, high-risk patients [43].

Emerging therapies, such as the combination of HMAs with venetoclax, have shown promise as potential bridging strategies. Early-phase clinical trials have demonstrated high response rates with these combinations, including in high-risk CMML and secondary AML, potentially providing a more effective means of reducing disease burden prior to allo-HCT [44, 45]. Novel oral formulations, such as decitabine/cedazuridine (ASTX727), have emerged to simplify outpatient management without compromising efficacy [46–49]. However, the use of these agents remains experimental in this setting, and further studies are needed to confirm their long-term impact on transplant outcomes.

Given the uncertainties surrounding the optimal pretransplant strategy, the current consensus among many experts is to prioritize early referral for allo-HCT without necessarily requiring pretransplant debulking, particularly for patients with aggressive disease features [21, 39]. This approach aims to reduce the risk of losing transplant eligibility due to disease progression or treatment-related complications.

### Donor Selection

Stem cell donor options for patients with CMML encompass both standard and alternative sources. Standard donors include HLA-matched siblings and matched unrelated donors (MUDs), while alternative options comprise haploidentical donors, mismatched unrelated donors, and, less frequently, unrelated umbilical cord blood [50–56]. The use of cord blood is limited by its lower cell dose and slower engraftment kinetics. Over the past two decades, advances in donor selection criteria, HLA typing, and transplant protocols have significantly broadened the donor pool available to CMML patients, paralleling progress in allo-HCT for other hematologic malignancies [57–59].

Comparative studies evaluating the impact of donor type on transplant outcomes have produced variable and sometimes conflicting results. Several analyses suggest that HLA-identical sibling donors may offer superior overall survival and lower relapse rates compared to MUDs, likely due to better immunologic compatibility and reduced risk of GVHD. However, other studies that adjust for confounding patient-specific variables–such as age, comorbidities, and disease status at the time of transplant–have found no statistically significant differences in outcomes between sibling and unrelated donors.

More recent data, particularly from large cohorts of patients with MDS, which are frequently used as a surrogate for CMML due to their overlapping biology, have introduced an important nuance:donor age appears to be a critical determinant of transplant success [60]. In these studies, younger MUDs were associated with improved disease-free survival and lower relapse rates compared to older HLA-matched siblings. This observation underscores the growing recognition that immunologic vigor and stem cell quality may outweigh the benefits of familial matching in certain clinical contexts.

Haploidentical donor allo-HCT has revolutionized transplant outcomes and access across hematologic malignancies [55]. While data for CMML remain scarce, most recent data found comparable results between haploidentical donor allo-HCT and matched related donors in CMML. However, of note, matched related donor allo-HCT was superior to haplodentical transplants for patients with CPSS lower-risk disease [61].

Furthermore, large-scale analyses have emphasized the significance of cytomegalovirus (CMV) serostatus compatibility [62–66]. CMV mismatches between donor and recipient have been associated with increased post-transplant complications and reduced survival. However, much of this data predates the widespread adoption of modern CMV prophylaxis agents such as letermovir, suggesting that the clinical impact of CMV mismatch may be evolving in the current therapeutic landscape.

Taken together, these findings highlight the need for a more individualized approach to donor selection in CMML. Rather than relying solely on donor relationship, transplant centers are increasingly incorporating donor age, performance status, CMV serostatus, and other biologic markers into donor selection algorithms. Future prospective studies specifically focused on CMML are needed to clarify these associations and guide optimal donor choice in this unique and complex patient population.

### Conditioning

Selecting the optimal conditioning regimen is a pivotal component of allo-HCT in patients with CMML [67, 68]. It involves a careful balance between the increased NRM risk associated with myeloablative conditioning (MAC) and the elevated risk of disease relapse seen with reduced-intensity conditioning (RIC) [69–71]. Current guidelines define MAC and RIC intensities based on data largely derived from MDS cohorts. However, retrospective studies in CMML have not consistently demonstrated the superiority of one approach over the other, with both MAC and RIC yielding comparable long-term survival in many settings.

Nevertheless, relapse rates in CMML post-transplant tend to be substantially higher than in MDS, with retrospective series reporting relapse in 27–52%of patients, and as many as 80%relapsing within the first year [12, 72]. These figures underscore the high relapse risk in CMML and suggest that, where clinically feasible, MAC may be preferable for fit patients, potentially offering better disease control and reducing early relapse.

For patients ineligible for MAC due to advanced age or comorbidities, alternative regimens such as fludarabine-busulfan (with or without total body irradiation) have shown promise in MDS and myeloproliferative neoplasms and may be extrapolated to CMML. More recently, treosulfan-based conditioning has gained traction due to its reduced toxicity profile and promising efficacy in hematologic malignancies, though direct evidence in CMML remains limited [67, 73–75].

In a retrospective study of 69 CMML patients undergoing allo-HCT, outcomes were compared across three conditioning regimens:thiotepa-busulfan (TB), sequential FLAMSA-busulfan fludarabine (FLAMSA-FB), and treosulfan-fludarabine (Treo-Flu). While engraftment times were comparable, the TB group demonstrated a notable advantage in 3-year progression-free survival (80%) and overall survival (80%) compared to FLAMSA-FB (33%and 37%) and Treo-Flu (39%and 55%). Importantly, there were no relapses in the TB cohort at 3 years, whereas relapse rates were 41%in FLAMSA-FB and 30%in Treo-Flu (*p* = 0.02). Rates of acute and chronic GVHD and NRM were similar across groups, positioning TB as a particularly promising conditioning strategy for CMML [73].

Another single-center retrospective study evaluated fludarabine-treosulfan (FT) versus fludarabine-busulfan with total body irradiation (FBT200) in a mixed cohort of MDS and CMML patients (*n* = 138) [76]. Using propensity score matching, the FT group exhibited significantly superior outcomes:2-year overall survival (66.9%vs. 44.5%, *p* = 0.013), relapse-free survival (63.1%vs. 39.1%, *p* = 0.008), and GVHD-free/relapse-free survival (57.4%vs. 35.1%, *p* = 0.02). One-year event-free survival and NRM were also more favorable in the FT group (40.3%vs. 9.2%, and 9.9%vs. 29.7%, respectively), supporting treosulfan as a viable alternative to busulfan, especially for patients at higher risk of toxicity.

A separate investigation explored the integration of a hypomethylating agent (HMA), decitabine, into the conditioning regimen alongside busulfan, fludarabine, cyclophosphamide, and cytarabine [77]. Among 48 patients (44 with MDS, 4 with CMML), this intensified regimen yielded an 86%survival rate at a median follow-up of 522 days, with a 12%relapse rate and 12%NRM. Severe acute GVHD occurred in 23%of patients, while chronic GVHD was reported in 15%. Notably, 2-year survival rates remained high even in patients with high-risk molecular profiles, including *TP53* and *ASXL1* mutations. These results suggest that integrating HMAs into conditioning may offer an effective disease control strategy for high-risk MDS and CMML patients, though further validation is warranted.

Collectively, these findings emphasize the importance of individualized conditioning regimen selection in CMML, taking into account patient fitness, disease risk, and molecular features to optimize post-transplant outcomes.

### GVHD Prophylaxis

A study analyzing the outcomes of 75 patients with MDS/MPN who underwent allo-HCT found that post-transplant cyclophosphamide (PTCy)-based GVHD prophylaxis was associated with improved survival outcomes [78]. Among the cohort, 71%received PTCy, and 59%received a combination of PTCy and ATG. At a median follow-up of 44.4 months, the 2-year rates were as follows:moderate-severe chronic GVHD (31.7%), NRM (37.9%), relapse (17.4%), GVHD-free/relapse-free survival (24.8%), and overall survival (51.6%). PTCy-based prophylaxis significantly improved survival (hazard ratio, 0.49, *p* = 0.03), NRM (hazard ratio, 0.41, *p* = 0.03), and GVHD-free/relapse-free survival (hazard ratio, 0.47, *p* = 0.009) without increasing relapse risk, suggesting that PTCy is an effective GvHD prevention strategy in this patient population.

**Fig. 3:**
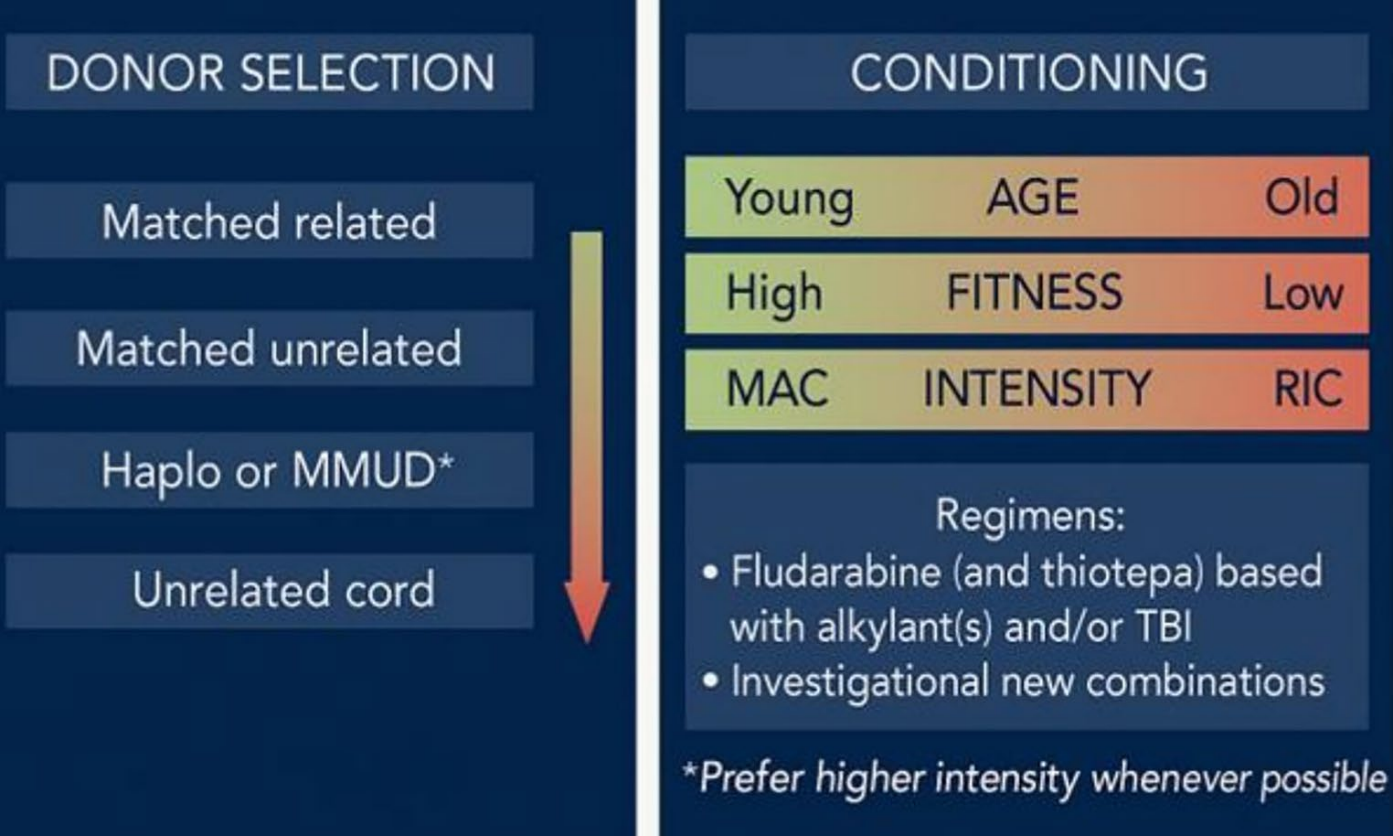
Donor and conditioning selection

However, while PTCy has significantly reduced GvHD, emerging data suggest it may be associated with a higher risk of disease relapse, particularly in the setting of reduced-intensity conditioning. This is thought to result from PTCy’s broad immunosuppressive effects, which may attenuate the graft-versus-leukemia response. The balance between GvHD prevention and maintaining effective graft-versus-leukemia remains a key challenge, especially in patients undergoing less intensive conditioning, where disease control is already more tenuous. Furthermore, its role in other donor transplants compared with ATG remains uncertain, and is most likely also affected by standard practice and access according to centers and approvals (Fig. 3) [79].

### Post-Transplant Monitoring

There are no contemporaneous studies on the role of MRD after allo-HCT in CMML. Findings have to be translated from studies in MDS or MPN [80–82]. Duncavage et al. [80] explored the potential of genomic profiling for MRD assessment in patients with MDS undergoing allo-HCT. Using enhanced exome sequencing of paired bone marrow and normal tissue samples, they identified patient-specific mutations, which were then tracked using error-corrected sequencing at 30 days post-transplant. Patients with detectable mutations at a variant allele frequency (VAF) of ≥ 0.5%at this early time point had a significantly higher risk of disease progression.

In a subsequent prospective study involving 266 MDS patients, the relationship between MRD and post-transplant relapse was further evaluated [83, 84]. Prior to transplant, patient-specific mutations were identified using a targeted 54-gene sequencing panel. Post-transplant, these mutations were monitored using droplet digital polymerase chain reaction (ddPCR), a highly sensitive technique with a detection threshold of 0.1%. Of the 44 patients who relapsed, 42 had detectable MRD in their bone marrow prior to clinical relapse, highlighting the strong correlation between MRD positivity and subsequent disease recurrence.

Based on these findings, an expert panel concluded that there is sufficient evidence to support the routine use of MRD monitoring in MDS patients after allo-HCT [85].

### Maintenance

While maintenance therapy after allo-HCT has shown limited benefit in MDS, the success of maintenance strategies in AML raises important questions about their potential role in CMML, which shares features with both MDS and myeloproliferative neoplasms, and relapse remains a leading cause of post-HCT failure. The disappointing results in MDS may partly reflect the typically lower proliferative burden and immunologically less dynamic disease environment, where maintenance therapy may not sufficiently alter the post-transplant trajectory. In contrast, AML–with its higher proliferation rate and molecular instability–may offer more therapeutic windows for maintenance to suppress minimal residual disease and prevent relapse.

In CMML, particularly the myeloproliferative subtype, biology may more closely resemble AML in terms of relapse risk, suggesting that selected patients could potentially benefit from post-HCT maintenance therapy. However, the heterogeneity of CMML makes one-size-fits-all strategies unlikely to succeed. Personalized maintenance approaches, potentially guided by molecular markers, MRD status, or CMML subtype, may hold greater promise. In short, while CMML has not yet been the focus of maintenance trials post-HCT, extrapolating from AML suggests that well-designed, biomarker-driven studies are warranted, especially for high-risk or proliferative cases where relapse risk is highest and the potential impact of maintenance may be most meaningful.

### Treatment of Relapse

The treatment of relapse remains a significant clinical challenge. Therapeutic strategies depend on the timing and nature of the relapse–whether molecular, hematologic, or overt–and must take into account patient fitness, previous therapies, and molecular risk factors. Hypomethylating agents such as azacitidine or decitabine are often re-employed in the post-transplant setting due to their immunomodulatory effects and tolerability, and may offer disease control, particularly in molecular or early hematologic relapse. For patients with targetable mutations, such as *IDH1/2* or *FLT3*, targeted therapies may provide an effective and less toxic alternative to conventional chemotherapy. Donor lymphocyte infusions (DLI) can also be considered in selected patients, especially those with evidence of minimal residual disease or mixed chimerism, although the risk of graft-versus-host disease must be weighed carefully. In relapsed cases not previously transplanted, allo-HCT remains the only potentially curative option and may be reconsidered if the patient is eligible [86–95]. For patients ineligible for intensive therapy, supportive care, clinical trial enrollment, or novel combinations–such as HMAs with venetoclax–may offer disease stabilization. Overall, the management of relapse requires a personalized approach, guided by disease kinetics, molecular profile, and patient-specific factors, with an emphasis on timely intervention and multidisciplinary coordination.

### Emerging Therapeutics and Clinical Trials

Recent research has expanded treatment options beyond traditional chemotherapy and transplantation. Targeted therapies such as venetoclax, IDH inhibitors, JAK2 inhibitors, and novel agents like tipifarnib are currently under clinical investigation. Immunotherapeutic strategies, including CAR-T cell therapies and bispecific antibodies, offer potential future treatment avenues, particularly for high-risk patients or those relapsing post-transplant [96, 97].

## Conclusion

Allogeneic transplantation remains the only curative option for CMML, significantly impacting long-term survival outcomes. However, careful patient selection, precise timing, optimal conditioning regimen choice, and rigorous post-transplant management are crucial to improving success rates. Ongoing research into novel therapies and improved risk stratification tools continues to refine transplantation strategies, offering hope for better outcomes and enhanced quality of life for patients with CMML.

## Key References

- (17) Tefferi A, Fathima S, Abdelmagid M, Alsugair A, Aperna F, Rezasoltani M, et al. BLAST:A globally applicable and molecularly versatile survival model for chronic myelomonocytic leukemia. Blood. 2025. e-pub ahead of print 20250507. 10.1182/blood.2024027170.
-
  - This study introduces the BLAST model, a globally applicable and molecularly informed prognostic tool that reflects the heterogeneity of CMML. It is a pivotal advancement for risk stratification and survival prediction, making it essential for guiding both therapeutic decisions and transplant eligibility.
- (25) Della Porta MG, Lannino L, Flamigni A, Hunter AM, Gagelmann N, Robin M et al. A decision support system for personalized optimization of hematopoietic stem cell transplantation timing in chronic myelomonocytic leukemia. EHA 2025:S176.
-
  - A novel, clinically relevant decision support system that personalizes transplant timing in CMML based on individual risk and disease dynamics.
- (42) Tremblay D, Csizmar C, DiNardo CD, Ball S, Rippel N, Hammond D et al. Venetoclax in combination with hypomethylating agents in chronic myelomonocytic leukemia:a propensity score matched multicenter cohort study. Leukemia 2025;39(1):257–260. e-pub ahead of print 20241112;10.1038/s41375-024-02466-6
-
  - One of the first multicenter studies evaluating venetoclax in combination with hypomethylating agents specifically in CMML. This work provides real-world evidence for emerging therapeutic strategies
- (52) Jain T, Tsai HL, Elmariah H, Vachhani P, Karantanos T, Wall SA et al. Haploidentical donor hematopoietic cell transplantation for myelodysplastic/myeloproliferative overlap neoplasms:results from a North American collaboration. Haematologica 2023;108(12):3321–3332. e-pub ahead of print 20231201;10.3324/haematol.2023.283426
-
  - A multicenter study demonstrating the feasibility and outcomes of haploidentical transplantation specifically in patients with myelodysplastic/myeloproliferative neoplasms, including CMML. It provides important data supporting the use of alternative donors in older or donor-limited patients.
- (73) Cassanello G, Serpenti F, Bagnoli F, Saporiti G, Goldaniga M, Cavallaro F et al. Treosulfan, thiotepa and fludarabine conditioning regimen prior to first allogeneic stem cell transplantation in acute myeloid leukemia and high-risk myelodysplastic syndromes:a single center experience. Bone Marrow Transplant 2023;58(9):1059–1061. e-pub ahead of print 20230624;10.1038/s41409-023-02023-2
-
  - Provides early data supporting the safety and efficacy of a treosulfan-thiotepa-fludarabine regimen in AML and high-risk MDS, which is often extrapolated to CMML. Conditioning intensity and regimen selection are central to transplant success, especially in older adults.
- (74) Pasic I, Moya TA, Remberger M, Chen C, Gerbitz A, Kim DDH et al. Treosulfan- Versus Busulfan-based Conditioning in Allogeneic Hematopoietic Cell Transplantation for Myelodysplastic Syndrome:A Single-center Retrospective Propensity Score-matched Cohort Study. Transplant Cell Ther 2024;30(7):681 e681-681 e611. e-pub ahead of print 20240420;10.1016/j.jtct.2024.04.014
-
  - A comparative study showing treosulfan-based conditioning may offer better toxicity and relapse profiles than busulfan in MDS. Given the overlap and frequent classification of CMML under MDS, these findings are highly relevant to transplant conditioning choices in CMML.
- (76) Desai N, Rodriguez-Rodriguez S, Chen C, Moya TA, Al-Shaibani E, Novitzky-Basso I et al. Outcomes of allogeneic hematopoietic stem cell transplantation for myelodysplastic/myeloproliferative overlap neoplasms. Eur J Haematol 2025 e-pub ahead Print 20250507;10.1111/ejh.14430
-
  - Evaluates the safety and outcomes of PTCy-based GVHD prophylaxis in CMML patients beyond the haploidentical donor setting.
- (94) Mesa RA. Tipifarnib:farnesyl transferase inhibition at a crossroads. Expert Rev Anticancer Ther 2006;6(3):313-319. doi:10.1586/14737140.6.3.313
-
  - Provides foundational insight into the development and rationale for farnesyl transferase inhibitors like tipifarnib, one of the few targeted therapies studied in CMML.
- Gagelmann N, Badbaran A, Beelen DW, Salit RB, Stolzel F, Rautenberg C, et al. A prognostic score including mutation profile and clinical features for patients with CMML undergoing stem cell transplantation. Blood Adv. 2021;5(6):1760–9. 10.1182/bloodadvances.2020003600.
-
  - The first comprehensive multicenter study encapsulating clinical, transplant-related and molecular data as risk factors for post-transplant overall survival and non-relapse mortality.

## Data Availability

No datasets were generated or analysed during the current study.
